# The Motives of Medical Reformists Exposed

**Published:** 1814-02

**Authors:** 


### *For the Medical and Physical Journal.*

### *The Motives of Medical Reformists exposed; by* SALUS PUBLICA.

Oh curas hominum ! oh quantum est in rebus inane !

THE public welfare!—the public welfare!—such is the cry of the geese who are to save the Capitol—while all who venture to dissent, who cannot apprehend the utility of the *patriotic* exertions of the London Committee, are branded as illiberal, their pens are sharpened by jealousy, and directed by avarice. These courteous terms are of course to be expected from men who arrogate to themselves every virtue under heaven; for, of consequence, how can any remain to their unfortunate adversaries? and the history of all controversy sufficiently shows, that where reason fails, the party are content to have recourse to abuse and aspersions—like Astolpho's lance, however, these cannot wound: and hence, careless of such opinions, like our paper currency, depreciated instead of valuable, I shall endeavor to offer a few more observations on the apothecaries' bill.

If I wanted arguments to strengthen my original opinion, that private interest was the primum mobile of all this *disinterested* clamour about medical reform, I could not deduce them from a better source than from the letters which its partizans have inserted in the Medical Journal; in almost all of these the physician is attempted to be degraded, and the honesty, the talents, the education of the surgeon-apothecary exalted to the seventh heaven. Not one of the tribe will allow but what his education is complete, and that he is perfectly qualified for the duties of his profession. The three years that *at least* must be spent by the candidate for medical honors, in comparative solitude and close application, the examination which at the end of that period *will prove* his acquirements,* are treated with apparent contempt; while five years of manual labor behind the counter, the mystery of rolling pills, the art of neatly labelling, with a six months' attendance upon the hospitals, are deemed amply sufficient to entitle these knights of the mortar to every degree of credit and reputation: like Dr. Ollapod they can "wing a pheasant and feel a pulse with e'er a man in the county." Such being the case, where is the necessity for a reform? Would it have been thought of, had not the prescriptions too frequently travelled to the druggist—hinc illæ lachrymæ! I hesitate not to express my sincere conviction that this measure is intended to establish the supremacy of the apothecaries; that it is hoped to render the physician

* Of course I speak at present of the most general school of instruction, Edinburgh.

subservient subservient to that branch of the profession, which from its numbers, its opportunities, and its habits, must generally come first into play, and necessarily acquire great influence; and that, if carried, the description of this vale of tears will no longer be considered as an allegory, nor the flight of Hygeia as a fable.

I verily believe that every human institution has a tendency to advance a certain degree towards perfection, but that all violent attempts to hurry its progress, will rather check than advance it. I am also perfectly convinced, that no theory, however ingenious, no paper plan, however apparently practicable, will ever prevent the progress of error, or the entrance of imperfection; time alone can work the change by imperceptible though powerful means. As to medical science, from its present advanced state, I see less occasion for reform than ever; yet far am I from saying it is not susceptible of improvement. None can lament more than myself that opprobrium of the profession, the St. Andrew's degrees; and yet I lament it more from the opportunities it affords of exciting reproach, than from its power of doing harm; those who adopt them are generally respectable apothecaries, who in the decline of life wish to avoid the more fatiguing branches of their profession; who feel themselves from long experience fully qualified for its higher departments, and who are thus able to superintend their younger and inexperienced brethren: to such men even I regret that our universities do not afford a readier admission. As to the name it sometimes affords to empirics, that is of little moment; as long as credulity and ignorance operate on the minds of men, so long will impostors start up and flourish. That little medical science is gleaned at our universities I readily grant, yet have I been surprised at the sneers cast upon them. The English degree is *honorable*, because it marks the man of general science and information. The students, out of their terms, are pursuing their medical studies in the hospitals of London or in the schools of Edinburgh, and those who know the light that the sciences mutually reflect upon each other, will agree with me, that a man thus educated is most likely to prove an ornament to his profession. As to the jokes cast upon us, "sunt nugæ," and pity were it, "si in seria ducent,"—'tis the fate of every profession, and of every one elevated beyond the sphere of common minds. Yet when the brilliancy of those who have obtained celebrity in wrangling courts, or reaped their honors in iron fields, shall have passed away, the enlightened observer of nature, the alleviator of human sufferings will be remembered and esteemed.

